# Bridging the gap: cancer scientific equity, global child health, and distribution of CAR T-cell therapy clinical trials in childhood cancer

**DOI:** 10.3389/fped.2025.1611187

**Published:** 2025-07-09

**Authors:** Kevin Fernando Montoya-Quintero, Johana Galván-Barrios, Darly Martinez-Guevara, Diana Dueñas, John Montenegro, Yamil Liscano

**Affiliations:** ^1^ Facultad de Ciencias Para la Salud, Universidad de Manizales, Manizales, Colombia; ^2^ Biomedical Scientometrics and Evidence-Based Research Unit, Department of Health Sciences, Universidad de la Costa, Barranquilla, Colombia; ^3^ Grupo de Investigación en Salud Integral (GISI), Departamento Facultad de Salud, Universidad Santiago de Cali, Cali, Colombia

**Keywords:** neoplasms, antineoplastic agents, clinical trials, global health, child, evidence gaps

## Abstract

Chimeric antigen receptor (CAR) T-cell therapy has transformed the treatment land-scape for childhood cancer. However, its global distribution remains unequal, with limited access in regions bearing a high burden of disease. This situation raises critical concerns about scientific equity in pediatric oncology research worldwide. To date, no study has systematically examined the scientific coherence between child health needs, global health indicators, and the frequency of CAR T-cell therapy clinical trials for childhood cancer. This omission represents a significant gap in the literature, with im-plications for global health equity and cancer research prioritization. A mixed-method analysis was conducted using global health metrics, child cancer indicators, and data from the Global Observatory on Health Research and Development. A total of 414 CAR T-cell therapy clinical trial participations across 30 countries were identified, with a heavy concentration in China (*n* = 161) and the United States (*n* = 84). High-income countries represented 73.3% of those participating. Multiple linear regression identified only one significant predictor for clinical trials participation: youth mortality (<15 years) (Coef. = 161.53; *p* = 0.045). The Lasso model revealed key predictors such as deaths due to alcohol use (Coef. = 29.99) and obesity (Coef. = 9.62) in children aged 5–14. Findings reveal a structural misalignment between childhood cancer disease burden and research activity in advanced therapies. Clinical trials are concentrated in countries with stronger scientific infrastructure rather than those with the greatest health needs, reinforcing cancer scientific inequities in the production and distribution of biomedical knowledge.

Despite remarkable progress in cancer research, childhood cancer continues to be a leading cause of death among children and adolescents worldwide (1). An estimated 400,000 new cases occur each year, yet significant disparities persist in access to innovation, research, and clinical development (1). Evidence shows that only 28% of child-hood cancer clinical trials are conducted in countries bearing 90% of the global disease burden (2), highlighting a critical misalignment between where innovation is generated and where it is most needed (2).

Chimeric Antigen Receptor (CAR) T-cell therapy represents one of the most promising advancements in pediatric oncology (3), offering curative potential for re-lapsed or refractory hematological malignancies (3). However, its global implementation remains limited. The complexity of manufacturing, high development costs, and structural inequalities in research ecosystems restrict the availability of CAR T-cell clinical trials to high-income countries (2), where the burden of disease is comparatively lower (2).

Understanding the current landscape in research distribution on CAR T-cell clinical trials in childhood cancer provides critical insights for policymakers, funding agencies, and global health stakeholders seeking to align innovation with real-world child health priorities (4). This issue aligns with World Health Organization (WHO)'s 2023 call for a more equitable research and development ecosystem in pediatric oncology (2), emphasizing the need for decentralized trial architecture, increased public investment, and innovative regulatory pathways (2). Furthermore, academic voices have highlighted the urgent need to adopt alternative models of development to ensure broader access and sustainability of cell therapies in pediatric populations (5, 6).

This scientific coherence has not been previously examined, representing a clear theoretical and population-level gap of global relevance. A specific analysis is therefore required to generate knowledge that can inform the academic and scientific communities, as well as global health decision-makers.

To explore this gap, a brief mixed-method scientometrics and global health metrics analysis was conducted, stratified by countries. The aim was to evaluate the scientific relevance and alignment between key health indicators, such as overall disease burden, childhood cancer burden, and major global risk factors for early-onset conditions, and the research and development landscape related to CAR T-cell therapy clinical trials for childhood cancer, as tracked by the WHO's Global Observatory on Health Research and Development (7). Data on the pipeline and landscape of childhood cancer drugs be-tween 2007 and July 2022 were collected (7), excluding entries marked as “unknown” by country to ensure the reliability of the dataset. The analysis focused specifically on clinical trials involving CAR T-cell therapy, categorizing products by their development phase and associated malignancy type.

Global health metrics were obtained from the United Nations World Population Prospects (8) and the WHO's Global Health Observatory (9), also stratified by countries. All metrics were extracted up to the most recently available year of data collection (Table 1).

**Table 1:** Global health, child health, health research, and cancer mortality metrics and indicators used in the statistical analysis.

Health indicator/metric	Definition	Metric year
HM-1	CHE as percentage of GDP (%)	2021
HM-2	Research and development expenditure (% of GDP)	2022
HM-3	Charges for the use of intellectual property, payments (BoP, current US$)	2023
HM-4	Health researchers (in full-time equivalent) per million inhabitants, by WHO region	2021
HM-5	Number of clinical trials by WHO region (1999–2022)	2023
HM-6	Population, ages 0–4	2023
HM-7	Population, ages 5–14	2023
HM-8	Population, ages 15–24	2023
HM-9	Death rate from cancer per 100,000 people	2021
HM-10	Death rate from air pollution per 100,000 people	2021
HM-11	Death rate from high blood sugar per 100,000 people	2021
HM-12	Death rate due to low physical activity per 100,000 people	2021
HM-13	Rate of deaths attributed to no access to handwashing facilities per 100,000 people	2021
HM-14	Death rate from obesity per 100,000 people	2021
HM-15	Death rate from pregnancy or maternal conditions per 100,000 people	2021
HM-16	Share of out-of-pocket expenditure on healthcare	2021
HM-17	Coverage of essential health services	2021
HM-18	Child mortality rate (< 5 years) (%)	2022
HM-19	Infant mortality rate (< 1 year) (%)	2022
HM-20	Neonatal mortality rate (< 28 days) (%)	2022
HM-21	Youth mortality rate (< 15 years) (%)	2022
HM-22	Child mortality by non-communicable diseases (deaths per 100,000 people)	2021
HM-23	Cancer deaths in children under five	2021
HM-24	Cancer deaths in children aged 5 to 14	2021
HM-25	Malnutrition: share of children who are underweight (%)	2023
HM-26	Malnutrition: share of children who are wasted (%)	2023
HM-27	Share of one-year-olds vaccinated against diphtheria, tetanus and pertussis (%)	2023
HM-28	Share of one-year-olds vaccinated against Haemophilus influenzae type B (%)	2023
HM-29	Share of one-year-olds vaccinated against hepatitis B (%)	2023
HM-30	Share of children fully vaccinated against measles (%)	2023
HM-31	Share of one-year-olds who are vaccinated against polio (%)	2023
HM-32	Share of one-year-olds vaccinated against rotavirus (%)	2023
HM-33	Share of one-year-olds vaccinated against rubella (%)	2023
HM-34	Share of one-year-olds vaccinated against Streptococcus pneumoniae (%)	2023
HM-35	Share of newborns vaccinated against tuberculosis (%)	2023
HM-36	Number of child deaths by urogenital congenital anomalies	2021
HM-37	Number of child deaths by congenital heart anomalies	2021
HM-38	Number of child deaths by digestive congenital anomalies	2021
HM-39	Number of child deaths by congenital musculoskeletal and limb anomalies	2021
HM-40	Number of child deaths by down syndrome	2021
HM-41	Number of child deaths by neural tube defects	2021
HM-42	Number of child deaths by orofacial clefts	2021
HM-43	Deaths by alcohol use for ages 5–14	2021
HM-44	Deaths by high blood pressure for ages 5–14 (%)	2021
HM-45	Deaths by poor sanitation for ages 5–14	2021
HM-46	Deaths by iron deficiency for ages 5–14	2021
HM-47	Deaths by no access to handwashing for ages 5–14	2021
HM-48	Deaths by high body-mass index (obesity) for ages 5–14	2021
HM-49	Deaths by high blood sugar for ages 5–14	2021
HM-50	Death rate from pregnancy or maternal conditions per 100,000 people	2021

BoP, current account balance; CHE, current health expenditure; GDP, gross domestic product; WHO, World Health Organization.

An inferential analysis was conducted using multiple linear regression to examine whether child health indicators predict the number of CAR-T cell therapy clinical trials in childhood cancer by country. Additionally, a Lasso (Least Absolute Shrinkage and Selection Operator) regression model, a regularized linear regression technique (10), was constructed to reduce the risk of overfitting and to better understand the key structural factors underlying the global distribution of advanced clinical trials. The selection of predictors was conducted using an exploratory, data-driven approach through cross-validated Lasso regression. All predictor variables were standardized prior to modeling to ensure comparability of coefficients. Each non-zero coefficient indicated a variable that significantly contributed to predicting the number of clinical trials. The magnitude of each coefficient represented the expected impact on the dependent variable for every standardized unit change in the predictor. All statistical analyses were performed using R (version 4.3.1).

A total of 414 cumulative participations in clinical trials were identified across 30 countries. The average number of clinical trials per country was 13.8 [Standard Deviation (SD) 31.8], although the distribution was highly skewed. The five countries with the highest number of studies were China (*n* = 161), the United States (*n* = 84), Italy (*n* = 20), the United Kingdom (*n* = 18), and France (*n* = 17). In contrast, 30% of the countries re-ported only one clinical trial. This pattern reflected a high concentration of clinical re-search within a limited number of countries.

Regarding geographic distribution, most countries were located in the European region (63.3%), followed by the Western Pacific (20%), the Americas (6.7%), South-East Asia (6.7%), and the Eastern Mediterranean (3.3%). In terms of income classification, high-income countries predominated (73.3%), followed by upper-middle-income countries (20%), and a small proportion of lower-middle-income countries (6.7%). No participation was observed from low-income countries or from countries in the African region. These findings demonstrated an inequitable global participation in research and development of advanced therapies for pediatric cancer.

Based on sponsor types, clinical trials were primarily initiated by academic and research institutions (*n* = 205; 69.2%; mean: 6.8 trials per country; SD 22.5), followed by the pharmaceutical and biotechnology industry (*n* = 83; 28.04%; mean: 6.5 trials per country; SD 9.3). In contrast, participation from public sectors (*n* = 5; 1.68%; mean: 0.1 trials per country; SD 0.5) and private non-industrial entities (*n* = 3; 1.01%; mean: 0.1 trials per country; SD 0.39) was marginal. These findings suggest the existence of a clinical development model globally driven by academic institutions and the pharmaceutical industry in this field.

Regarding clinical trial phases, early-phase studies predominated, with phase I trials accounting for the majority (*n* = 26; 54%; mean: 1.27; SD 3.53), followed by phase II trials (*n* = 20; 42%; mean: 1.57; SD 3.61). Minimal representation was observed for phase III or commercialization-stage trials. This finding indicates that most studies remain in early stages of development, which may be related to the highly experimental nature of CAR-T cell therapies in the pediatric population.

Regarding the drugs under investigation by cancer category, 51.5% (*n* = 33/64) were related with leukemia, followed by 25% (*n* = 16/64) related to solid tumors. Lymphomas and brain tumors accounted for 12.5% (*n* = 8/64) and 10.9% (*n* = 7/64), respectively, of the therapeutic targets. Drugs were also identified across multiple subcategories of specific tumor types, with acute myeloid leukemia (*n* = 22/129; 17.05%), acute lymphoblastic leukemia (*n* = 13/129; 10.07%), and synovial sarcoma (*n* = 11/129; 8.52%) representing the most frequently studied malignancies. Anaplastic large cell lymphoma and retinoblastoma were the least frequently targeted malignancies, with two drugs under investigation for each.

The analysis of 50 indicators related to child health, global health, and population health revealed substantial heterogeneity across countries. Although specific values varied by variable, countries with the highest number of clinical trials were found to correspond with those reporting more favorable metrics such as mortality rates from chronic diseases, mortality attributable to chronic disease risk factors, coverage of essential health services, health expenditure, and child/youth mortality. Significant data dispersion was observed across multiple indicators, reinforcing the existence of a structural gap between countries with high clinical research capacity and those with greater public health needs.

Countries with the frequency of clinical trials (top 10) consistently demonstrated better averages in childhood health indicators. For example, the average under-five mortality rate among these countries was 0.42, compared to an average of 0.57 among the 10 countries with the fewest clinical trials. The global average under-five mortality rate was 0.52 (SD: 0.51), potentially reflecting a lower burden of childhood disease in the most research-active countries.

Indicators such as the infant mortality rate (<1 year) and neonatal mortality rate (<28 days), potentially linked to nutritional conditions or access to health services, also showed lower values in countries with a higher number of clinical trials (averages of 0.34 and 0.24, respectively) compared to those with fewer trials (averages of 0.49 and 0.32, respectively). This disparity suggests that scientific activity is not concentrated in regions with the greatest pediatric health needs, but rather in those with more favorable health conditions.

The indicator for child mortality due to non-communicable diseases (deaths per 100,000 population) showed a global average of 30.9 (SD 15.9), with a lower mean observed in countries with high research activity (mean 28.9) and a higher mean in countries with low research activity (mean 34.3). This finding may indicate that research efforts are being carried out in contexts where health systems already provide adequate coverage, rather than in settings with the most pressing deficits.

The linear regression model identified only one statistically significant indicator. The youth mortality rate (<15 years) exhibited a coefficient of 161.53 (95% CI: 4.18–318.89; *p* = 0.045), suggesting that a one-unit increase in this indicator was associated with an average increase of 161 clinical trials (Figure 1A). Although not representative of the overall trend, this finding indicates that higher mortality in individuals under 15 years of age is associated with a greater frequency of clinical trials. However, it must be noted that China and the United States, countries with the highest under−15 mortality rates among those with the greatest number of clinical trials, accounted for 59.1% of all global trial participations, potentially skewing the global trend.

**Figure 1:**
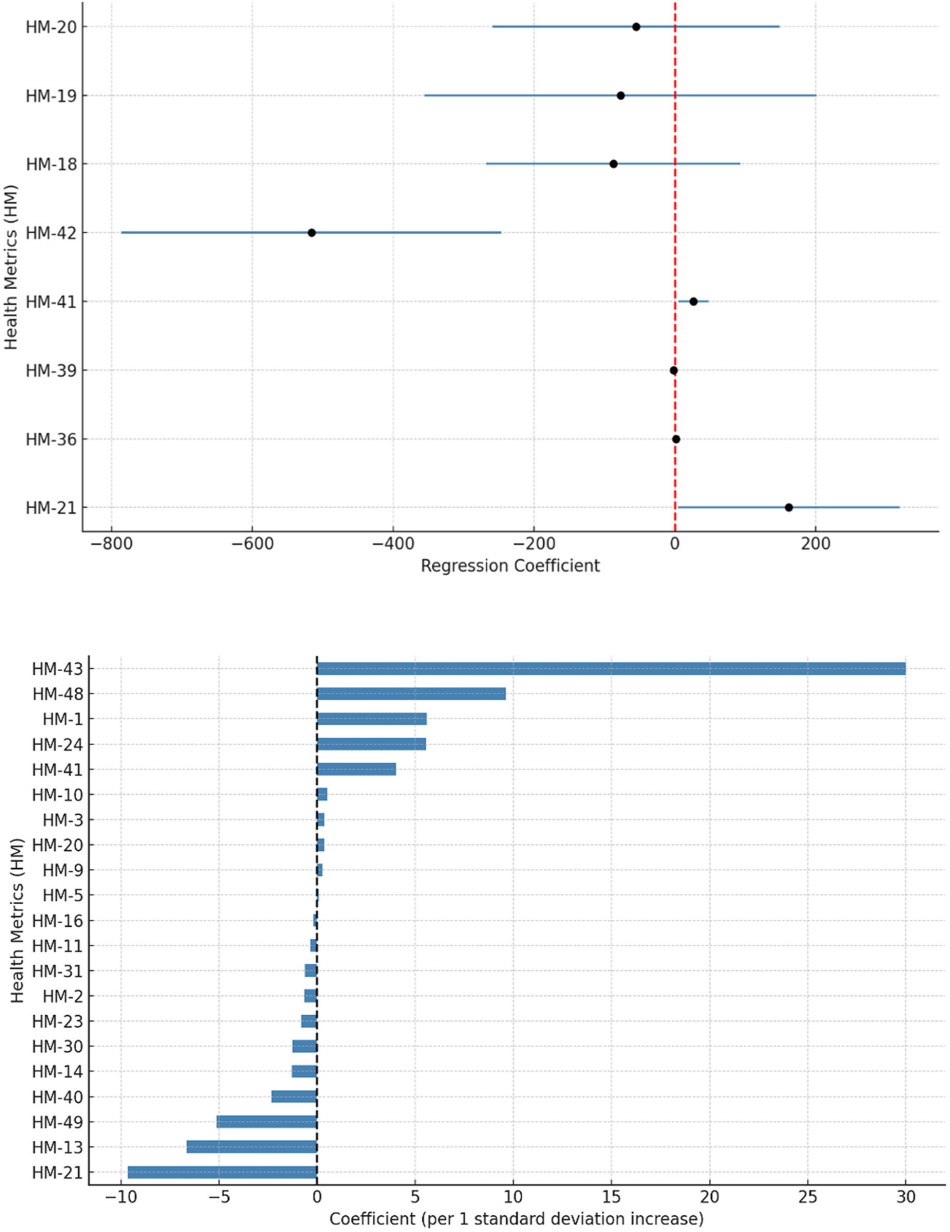
**(A)** Forest plot of the linear regression model, displaying only the metrics selected based on the following criteria: (1) statistical significance (*p* < 0.05); or (2) notable coefficients (≥ ± 50). **(B)** Distribution of health indicator coefficients with potential predictive value for the frequency of clinical trials, according to the Lasso model. Each one-standard-deviation increase in a given indicator is associated with either a higher or lower frequency of CAR T-cell therapy trials for childhood cancer by country. Definitions of each metric can be found in Table 1.

Other globally relevant metrics, including the child mortality rate (< 5 years) (Coef. = –87.56; 95% CI: −268.6 to 93.5; *p* = 0.317), infant mortality rate (<1 year) (Coef. = –77.25; 95% CI: −355.5 to 201.0; *p* = 0.561), and neonatal mortality rate (<28 days) (Coef. = –55.07; 95% CI: −258.7 to 148.5; *p* = 0.571), did not show statistically significant associations. Although no significant effects on the number of trials were demonstrated, the negative coefficients suggest that countries with poorer child health indicators tend to conduct fewer trials (Figure 1A), which aligns with the patterns observed in the descriptive analysis.

To identify the most relevant predictors of the number of CAR-T cell therapy clinical trials for childhood cancer by country, a Lasso regression model with cross-validation was applied. This approach is particularly useful in contexts with a high number of predictors and a limited sample size (*n* = 30 countries), as it enables automatic variable selection by retaining those with the greatest explanatory power while omitting redundant or irrelevant variables.

The model included all global health metrics and indicators as independent variables. The analysis identified five variables with non-zero coefficients, indicating that they contributed to the predictive model and were significantly associated with variation in the number of clinical trials per country.

The variable with the greatest predictive weight was deaths due to alcohol use in children aged 5–14 years (Coef. = 29.99). The second most relevant predictor was deaths due to high body mass index (obesity) in the same age group (Coef. = 9.62). Additional predictors included current health expenditure as a percentage of gross domestic product (Coef. = 5.59), cancer-related deaths in children aged 5–14 (Coef. = 5.55), and the number of child deaths due to neural tube defects (Coef. = 4.00) (Figure 1B). The presence of positive coefficients suggests that greater research activity in advanced therapies is observed in contexts where these conditions are more prevalent or more accurately reported.

Notably, the variable most strongly retained by the Lasso model, alcohol-attributable mortality among children aged 5–14, refers to deaths in which the child's own alcohol consumption is considered a contributing cause. While such cases are rare, their presence reflects environments where children are exposed to alcohol at an unusually early age. This early initiation often coexists with permissive social norms, inadequate regulatory oversight, and marked socioeconomic hardship (11), factors that also hinder the infrastructure and capacity needed to support complex, high-cost re-search such as CAR T-cell trials. For this reason, we interpret this indicator not as a direct driver of trial distribution, but rather as a sentinel of deeper, structural vulnerability.

The Lasso model automatically excluded multiple indicators related to child mortality, malnutrition, risk factors for chronic diseases in childhood (including cancer), and other variables typically associated with high pediatric disease burden or deficient public health conditions. This suggests that such indicators did not contribute to the predictive model of research activity, thereby reinforcing the hypothesis of a disconnect between healthcare need and the generation of scientific evidence.

These findings reveal a structural pattern: countries leading clinical research in CAR T-cell therapy for childhood cancer are those with stronger overall health indica-tors, higher public investment in health, broader coverage of essential services, and greater capacity in health-related science and technology. In other words, countries with the highest need for therapeutic advances due to a substantial burden of childhood cancer are not necessarily engaged in the clinical development of new technologies for pediatric cancer treatment.

The results suggest a lack of clear alignment between the distribution of clinical trials on CAR T-cell therapy for childhood cancer, scientific equity, and global child health. On the contrary, research activity is concentrated in high-income countries and specific regions, which may reflect structural, economic, and scientific barriers to conducting such studies in settings with greater disease burden and more limited health resources. This asymmetry underscores a significant gap in scientific and global health equity (12–14), where populations with the greatest needs are not necessarily the first to benefit from biomedical innovation, despite being the most vulnerable to childhood diseases (15, 16).

Drawing from the WHO's frameworks for equitable access to cell- and gene-based therapies (2), as well as the proven impact of pediatric oncology twinning programs, we propose a coordinated strategy to help close the research gap. At the heart of this approach lies a network of North–South and South–South partnerships that connect high-volume centers with emerging institutions, fostering the exchange of protocols, joint participation in virtual oncology boards, and access to shared biostatistical resources. These collaborations would be supported by regional hubs for good manufacturing practices, established through technology transfer agreements, enabling academic labs in low- and middle-income countries to produce CAR vectors locally at significantly lower costs than those of commercial suppliers.

Addressing regulatory and capacity-building challenges is equally essential. Mechanisms such as the WHO Collaborative Registration Procedure (17) and joint scientific consultations between established and developing regulatory agencies could substantially shorten dossier review times (17). Meanwhile, low- and middle-income countries-led seed grant programs, with protected budgets for pharmacovigilance, could catalyze early-phase, locally anchored trials. The use of adaptive platform trial designs, including shared control arms and Bayesian borrowing, would help lower the per-patient cost of research. In parallel, investments in training and retention, through fellowships in cell processing, bioinformatics, and pharmacoeconomics, along with incentives to keep talent in-country, would strengthen long-term capacity. Taken together, these interdependent actions offer a viable path toward reducing the research inequities highlighted in this study.

One important limitation to acknowledge is that some of the predictors retained by the model, such as alcohol-attributable deaths among children aged 5–14, are better understood as sentinel indicators of underlying social vulnerability, rather than as direct mechanistic determinants of how research activity is distributed. Similarly, it is important to highlight the limitation posed by missing data within the database, which restricts the accuracy of country-specific statistical results. Nevertheless, due to the nature and small size of the sample of recorded countries, addressing the issue of missing data remains challenging. Another limitation is the potential underreporting of trials in registries, especially those in early phases or not industry-sponsored. Moreover, the absence of qualitative data from low- and middle-income countries stakeholders limits the contextual interpretation of research barriers.

This analysis contributes to the understanding of global imbalances in research and development (18–20), offering an evidence-based foundation to inform future strategies aimed at aligning clinical innovation with public health priorities in experimental clinical research in childhood cancer (21). Addressing this disparity requires collaborative efforts to strengthen research capacity in underrepresented regions, integrate academic and regulatory initiatives, and ensure that novel therapies reach the populations most in need (22–24).

## Data Availability

The original contributions presented in the study are included in the article/Supplementary Material, further inquiries can be directed to the corresponding author.
